# A performance comparison of eight commercially available automatic classifiers for facial affect recognition

**DOI:** 10.1371/journal.pone.0231968

**Published:** 2020-04-24

**Authors:** Damien Dupré, Eva G. Krumhuber, Dennis Küster, Gary J. McKeown

**Affiliations:** 1 Business School, Dublin City University, Dublin, Republic of Ireland; 2 Department of Experimental Psychology, University College London, London, England, United Kingdom; 3 Department of Mathematics and Computer Science, University of Bremen, Bremen, Germany; 4 Department of Psychology and Methods, Jacobs University Bremen, Bremen, Germany; 5 Department of Psychology, Queen’s University Belfast, Belfast, Northern Ireland, United Kingdom; University of Colorado Boulder, UNITED STATES

## Abstract

In the wake of rapid advances in automatic affect analysis, commercial automatic classifiers for facial affect recognition have attracted considerable attention in recent years. While several options now exist to analyze dynamic video data, less is known about the relative performance of these classifiers, in particular when facial expressions are spontaneous rather than posed. In the present work, we tested eight out-of-the-box automatic classifiers, and compared their emotion recognition performance to that of human observers. A total of 937 videos were sampled from two large databases that conveyed the basic six emotions (happiness, sadness, anger, fear, surprise, and disgust) either in posed (BU-4DFE) or spontaneous (UT-Dallas) form. Results revealed a recognition advantage for human observers over automatic classification. Among the eight classifiers, there was considerable variance in recognition accuracy ranging from 48% to 62%. Subsequent analyses per type of expression revealed that performance by the two best performing classifiers approximated those of human observers, suggesting high agreement for posed expressions. However, classification accuracy was consistently lower (although above chance level) for spontaneous affective behavior. The findings indicate potential shortcomings of existing out-of-the-box classifiers for measuring emotions, and highlight the need for more spontaneous facial databases that can act as a benchmark in the training and testing of automatic emotion recognition systems. We further discuss some limitations of analyzing facial expressions that have been recorded in controlled environments.

## Introduction

The ability to accurately detect what other people are feeling is an important element of social interaction [1]. Only if we can perceive the affective state of an individual, will we be able to communicate in a way that corresponds to that experience. In the quest for finding a ‘window to the soul’ that reveals a view onto another’s emotion, the significance of the face has been a focus of popular and scientific interest alike. Since the publication of Charles Darwin’s book *The Expression of the Emotions in Man and Animals* [2], facial behavior has been considered to play an integral role in signaling emotional experience. According to Darwin, facial movements became associated with emotions as biological remnants of actions that once served survival-related purposes [3]. Whilst he did not postulate an intrinsic link between emotions and facial expressions, his work became fundamental to the emotion-expression view of Basic Emotion Theory (BET). Originally proposed by Tomkins [4], BET assumes that there are a limited number of emotions (e.g., happiness, sadness, anger, fear, surprise, and disgust) that are characterized by signature expressions [5, 6]. The emotions with which these expressions are associated are claimed to be basic, primary, or fundamental in the sense that they form the core emotional repertoire [6, 7]. Facial behavior, accordingly, has been seen as a “readout” [8] of these subjective feeling states, comprising specific configurations of facial muscle actions that are prototypical, innate, and universal.

In recent years, the traditional view that certain emotional states are signaled by a matching facial expression has been challenged. Even though BET has obtained popular support [1], evidence for a unique emotion-expression link is inconclusive [9]. As such, it is possible for an individual to feel an emotion without expressing it. Alternatively, not every facial expression may communicate an affective state [10, 11]. Debates about the role and function of facial movements have led to alternative frameworks such as the social constructivist approach [12–17]. In this view, faces are best conceived of as tools displaying signals in social interaction that can vary across cultures, situations, and individuals [18]. Although contemporary views of emotion consider facial activity within a rich set of socio-cultural and contextual factors, BET has been so far the primary focus of scientific research.

Inspired by the vision of an emotionally intelligent machine, efforts have been targeted towards computer systems that can detect, classify, and interpret human affective states. This involves the ability to recognize emotional signals that are emitted by the face [19, 20], post-hoc from video recordings as well as in real-time from a live stream camera [21]. In the wake of rapid advances in computer vision and machine learning, competing computational approaches now exist that focus on the analysis of facial expressions. Automatic facial affect recognition has significant advantages in terms of time and labor costs over human coding [22] and has been envisioned to give rise to numerous applications in fields as diverse as security, medicine, education, telecommunication, automotive, and marketing industries [23, 24]. While the computational modelling of emotional expressions forms a narrow, although increasingly common, approach, the ultimate aim is to build human-computer interfaces that not only detect but also respond to emotional signals of the user [25, 26]. To this end, computer algorithms generally follow three steps in classifying emotions from human facial behavior. First, they identify and track one or more faces in a video stream based on morphological features and their configuration. Second, they detect facial landmarks and evaluate their changes over time. Finally, they classify the configuration of landmarks according to specific labels, categories, or dimensions [27]. It is within the context of the last step where BET has exerted a profound impact on how expressive behavior is analyzed. Despite inconclusive scientific evidence in support the BET [9], most computer models have adopted its perspective by focusing on the six basic emotions [28, 29]. That is, they output a categorical emotion label from a limited set of candidate labels (i.e., happiness, sadness, anger, fear, surprise, and disgust), derived from the assumption that emotional expressions correspond to prototypical patterns of facial activity [7].

In the last three decades, substantial progress has been made in the area of automated facial expression analysis by recognizing BET’s six categories. Zeng, Pantic, Roisman and Huang [30], for example, reviewed 29 vision-based affect detection methods, pointing towards the proliferation of programs and platforms that are concerned with classifying distinct emotions. As demonstrated by the first Facial Expression Recognition and Analysis (FERA) challenge, emotion recognition by the top performing algorithm was already being reported in 2011 at a rate of 84% [31]. Together with recent news reports that forecast a bright future for emotionally intelligent machines [32, 33], the impression arises that the automatic inference of basic emotions may soon be a solved problem [34]. The majority of past efforts, however, relied on in-house techniques for facial affect recognition. As such, they involve classification algorithms that have been developed and benchmarked in individual laboratories, often using proprietary databases of emotion-related images and videos. Historically, those were not easily accessible for systematic interdisciplinary and cross-laboratory research. Given that automated methods for measuring facial expression patterns have now matured, 16 providers of commercially available classifiers have recently been identified [35, 36]. These classifiers are marketed for monitoring and evaluating human affective states across a range of domains. As a consequence, their performance can be assessed more freely and openly. Interestingly, however there exists little validation research that has investigated the overall and relative performance of these automatic classifier.

In a study by Lewinski, den Uyl and Butler [37], the commercial FaceReader classifier (VicarVision) was tested on static facial images of posed expressions, achieving a recognition rate of 89%. Using similar sets of static basic emotion stimuli, Stöckli et al. [38] reported performance indices of 97% and 73% for Facet (Emotient) and Affdex (Affectiva), respectively. While Facet was found to exceed human judges in classifying emotions on these standardized sets of static emotional portrayals, its accuracy dropped to 63% for dynamic stimuli depicting real-life facial expression imitations. A performance index of 80% was recently reported using FaceReader in the context of dynamic expressions that were enacted to also mimic a basic emotion display [39]. When testing the software CERT (a precursor of Facet) on subtle dynamic (i.e., non-prototypical) facial stimuli, Yitzhak et al. [40] found that emotion classification accuracy for subtle expressions (21%) was significantly reduced in comparison to highly intense and stereotypical expressions (89%). Such a large performance drop did not occur for human observers (79% vs. 88%), who were able to identify the relevant emotion expression in the absence of prototypical facial movements. Importantly, none of the above studies examined emotion recognition in spontaneous affective displays.

Given that there are fundamental differences between posed and spontaneous stimuli in their appearance and timing [41], it is important to draw a distinction between the two expression types. Spontaneous displays (similar to posed ones) may occur in a controlled setting (often in the laboratory), but the resulting emotional expression is induced (i.e., via presentation of emotionally laden pictures/movies) rather than instructed [42]. As such, they have distinct temporal and morphological profiles due to differences in emotion elicitation; the technical features (e.g., camera angle, head movement, illumination) remain largely the same. Subjecting only deliberately displayed expressions to automatic classification, analysis, and benchmarking may provide insufficiently robust validation results. Consequently, affective analyses based on deliberate and often prototypical displays are likely to be substantially less reliable with respect to spontaneous expressive behavior. This issue is further exacerbated by the general trend to train computer algorithms on posed expressions that are highly intense and homogeneous [43]. The third step in automated facial expression analysis typically involves a training set of human-labelled stimuli to make inferences about a much larger population of faces and facial expressions in which they occur [30]. Unless a computer system is validated on posed as well as spontaneous facial actions, its use in the public and private sector will likely prove inadequate. As the affective computing market is projected to grow considerably, with growth estimations reaching $41 billion by 2022 [44] and $90 billion by 2024 [45], a systematic multi-system evaluation of commercial automatic classifiers using both types of emotional expressions is needed.

The present research aims to fill this gap by testing 8 commercially available automatic classifiers and comparing their recognition performance to human observers. To this end, facial stimuli were sampled from two large databases that depict emotions either in a posed or spontaneous form. All of the examined expressions are dynamic to reflect the realistic nature of human facial behavior [46, 47]. Following common approaches in the development of these classifiers, itself a contentious issue beyond the scope of this article, we focused on the recognition of the six basic emotions identified by BET.

To assess the emotional content of expressions, participants selected the emotion label that best fits with a stimulus (forced choice). We predicted the classification accuracy of posed stimuli to exceed that of spontaneous ones, with generally reduced performance of the automatic classifiers compared to human observers in the context of spontaneously occurring expressions. Given the predominance of posed datasets for the training of classifiers, confusion patterns found for automatic classification should be more similar to those produced by human observers when analyzing deliberate affective displays.

## Materials and methods

For the present research, two well-known dynamic facial expression databases were chosen: *BU-4DFE* [48] and *UT-Dallas* [49]. Both are annotated in terms of emotion categories, and contain either posed or spontaneous facial expressions. To evaluate the accuracy of emotion recognition, we compared the performance achieved by human judges with those of 8 commercially available automatic classifiers. To this end, we first conducted a judgment study with naive human observers. Second, we assessed the performance of the automatic classifiers on the same databases, and employed standard metrics for all human versus automatic classifier-based comparisons.

### Stimulus material

Based on a recent review of 22 dynamic facial expression databases [50], we selected two datasets that are publicly available to the research community. *BU-4DFE* and *UT-Dallas* both contain large amounts of videos portraying the six basic emotions. Besides conceptual differences in elicitation method and thematic approaches, stimuli from the two databases are similar in the sense that they depict frontal head shots at close distance with comparable expressive intensity envelopes, a static camera view, and adequate illumination. All videos are rendered in color and captured with a frame-rate of 25 frames per second. While *BU-4DFE* contains particularly high-resolution video data (1094x1392; *UT-Dallas*: 720x480), both provide adequate resolution for facial analysis that meets the expected requirements for automatic classification [50].

The *BU-4DFE* database contains videos of posed expressions recorded from 78 individuals. They represent male and female subjects, mostly undergraduates, graduates and faculty members with an age range of 18-45 years, recruited from the State University of New York at Binghamton, USA. The majority of subjects are White, although, the database includes some Asians, Blacks, and Hispanics. Each subject was instructed by a psychologist to gradually portray the six basic emotions in distinct sequences. As one video is missing from the database, a set of 467 videos was processed: anger (78), disgust (78), fear (78), happiness (78), sadness (78), and surprise (77). Expression sequences lasted on average 4s (*M* = 4.05, *SD* = 0.43), and started and ended with a neutral face.

The *UT-Dallas* database is substantially larger and consists of videos of spontaneous expressions recorded from 292 individuals and a total of 961 videos with basic emotion labels recorded from different camera angles. They represent male and female students with an age range of 18-25 years, recruited from the University of Texas at Dallas, USA. The majority of subjects are White, including some Asians, Blacks, and Hispanics. Each subject watched a 10-minute video that included scenes from different movies and television programs intended to elicit distinct emotions. Selected emotive instances were extracted by the database authors, with expressive behavior corresponding to the six basic emotions. Given the lack of any validation data for this database, the assignment of a video to an emotion category reflects the subjective judgment of the database authors. We selected the first out of two sets (up to participant ID 4660) from the database to obtain a stimulus set of comparable size. This resulted in a total of 470 videos with an uneven amount of videos per emotion category: anger (3), disgust (119), fear (13), happiness (196), sadness (38), and surprise (101). Given the complex nature of spontaneous behavior, videos can include more than one type of facial expression [49]. Spontaneous expressions lasted on average 6s (*M* = 6.11, *SD* = 0.68), and started/ended with a neutral or expressive face. For a comprehensive review of both databases, readers are referred to [50].

### Human observers

Fourteen participants (10 females, *M*age = 24.0, *SD* = 6.62), recruited via email from the academic community in Germany, Turkey, and the UK, volunteered to participate for free or a monetary reward in an online study. The study was approved by the departmental ethics committee at University College London, UK. Informed consent was obtained prior to participation. Data management and data treatment were performed under the European GDPR legislation. Participants were told that short videos of facial expressions would be presented. Their task was to indicate the label which best described the displayed expression. They were instructed to watch all 937 videos attentively and with sufficient rest periods. Videos were shown in an individually randomized order and with scrambled file names to avoid guessing of the correct labels.

In line with common categorization paradigms, emotion recognition was assessed through a forced-choice task. This required participants to make a selection among the following emotion labels: *anger*, *disgust*, *fear*, *happiness*, *sadness*, *surprise*, *no/other emotion*. We opted for this response format to allow for direct comparability with the automatic classifiers’ recognition data using pre-specified emotion labels. As shown in prior research, adding a *no/other emotion* escape option does not change the overall level of target emotion recognition [51]. Instead, it only prevents agreement on incorrect labels when the target emotion label is absent [52].

In addition to the standard classification task, participants were asked to evaluate each video on perceived genuineness of the expressed emotion, using a 7-point Likert scale (1 -*very posed*, 7 -*very genuine*). An expression was defined as genuine if the person is truly feeling the emotion, in contrast to a posed expression which is simply put on the face in the absence of a corresponding emotion. Results showed that participants judged posed expressions as significantly less genuine than spontaneous ones (BU-4DFE: *M* = 3.42, *SD* = 1.79; UT-Dallas: *M* = 4.6, *SD* = 1.81; *t*(13, 023) = −37.39, *p* < .001, *d* = 0.66), thereby validating the two different emotion elicitation approaches for database construction.

### Automatic classification

The 937 video stimuli (467 BU-4DFE, 470 UT-Dallas) were submitted to automatic facial expression analysis by the following eight automatic classifiers: Affectiva’s Affdex, CrowdEmotion’s FaceVideo, Emotient’s Facet, Microsoft’s Cognitive Services, MorphCast’s EmotionalTracking, Neurodata Lab’s EmotionRecognition, VicarVison’s FaceReader and VisageTechnologies’ FaceAnalysis. These automatic classifiers can be used either through an Application Programming Interface (API), a Software Development Kit (SDK) or a software platform. All of them offer a prototypical basic emotion approach by classifying facial expressions in terms of the basic six emotions (anger, disgust, fear, happiness, sadness, and surprise).

*Affdex* (SDK v3.4.1) was developed by Affectiva which is a spin-off company resulting from the research activities of the MIT Media Lab created in 2009 [53]. At present, it is distributed by Affectiva (API and SDK) as well as iMotions (SDK integrated in a software platform). Affdex’s algorithm uses Histogram of Oriented Gradient (HOG) features and Support Vector Machine classifiers for facial expression recognition [54].

*FaceVideo* (API v1.0) was developed by the company CrowdEmotion founded in 2013. Its algorithm uses Convolutional Neural Networks, allowing the recognition of the six basic emotions plus neutral.

*Facet* (SDK v6.3) was originally developed by Emotient and distributed by iMotions in its software suite. Initially a spin-off company by the University of California San Diego [55], Emotient was bought by Apple Inc. in 2017. For this reason, Facet is no longer commercially available, but existing licences are still supported by iMotions.

*Cognitive Services: Face* (API v1.0) was developed by the company Microsoft on its Azure platform and first released in 2015. It provides a suite of artificial intelligence tools for face, speech, and text analysis.

*EmotionalTracking* (SDK v1.0) was developed by the company MorphCast founded in 2013. EmotionalTracking SDK is a JavaScript engine requiring less than 1MB, that works directly on mobile browsers (i.e, without remote server and API processing).

*EmotionRecognition* (API v1.0) was developed by the company Neurodata Lab founded in 2016. Neurodata Lab provides a suite of tools for emotion recognition or annotation experiments such as face recognition, speaker diarization, body pose estimation, heart rate and respiration rate tracking. Neurodata Lab’s EmotionRecognition is available both in API and SDK.

*FaceReader* (software v7.0) was developed by VicarVison and is now distributed by Noldus [37]. Initially presented in 2005 [56], the software uses Active Appearance Models for face modelling and Convolutional Neural Networks for facial expression classification [57]. All default settings were used for the video processing.

*FaceAnalysis* (SDK v1.0) was developed by the company Visage Technologies founded in 2002. Visage Technologies provides solutions for facial expression recognition as well as for ID verification using face recognition.

For all computer-based systems, performance indicators as reported in the present research are based on the respective version indicated above. Results may be subject to change with the release of newer versions. Because the type of output is not exactly the same in each system, emotion recognition results were rescaled to the odds ratios of recognition probability ranging from 0 to 1.

### Data analysis

The data analysis focuses on a comparison in emotion recognition performance between human observers and each of the eight automatic classifiers. It is important to note that classification outputs differ slightly between humans and the machine. While human observers are selecting an emotion label per video, automatic classifiers are providing a recognition odds ratio for every emotion label frame by frame. Therefore, two separate metrics were employed to identify the emotion recognized based on the calculation of a confidence score.

For the human observer data, the emotion recognition index corresponds to the emotion with the largest human confidence score among the six emotion labels (i.e., the label chosen by the highest number of human observers). As such, the number of correctly classified videos within an emotion category is divided by the total number of videos per emotion category aggregated across all human observers. The process to determine the recognized emotion label follows the Eq (1) for each video:

$$\begin{array}{c} EmoRec_{i,j}=\max(\frac{1}{K}\sum_{k=1}^{K}EmoRec_{i,j,k}) \end{array}$$ (1)

where *i* is a judged video, *j* is a category of emotion recognized (EmoRec), *k* is the number of human observers choosing the label *j*, and *K* is the total number of human observers for the video *i*.

In the context of the automatic classifiers’ data, the emotion recognition index corresponds to the emotion with the highest recognition confidence score among the six emotion labels. As such, it reflects the number of videos within an emotion category for which a given automatic classifier correctly indicated the highest recognition confidence score, divided by the total number of videos per emotion category. The automatic recognition confidence score [58] corresponds to the sum of the odds ratios for a specific emotion (e.g., happiness) aggregated per video-frame relative to the sum of the odds ratios for all other emotions (e.g., anger, disgust, fear, sadness, surprise) [58]. The process to determine the recognized label follows the Eq (2) for each video:

$$\begin{array}{c} EmoRec_{i,j}=\max(\frac{\sum_{x=0}^{T}\psi_{x,i,j}}{\sum_{j=1}^{J}\sum_{x=0}^{T}\psi_{x,i,j}}) \end{array}$$ (2)

where *i* is a processed video, *j* is a category of emotion recognized (EmoRec), *t*_*x*_ corresponds to the timestamp of the processed video and *ψ*_*x*,*i*,*j*_ the value of the odds ratio for the frame *t*_*x*_ and for the emotion label *j* such as *ψ*_*x*,*i*,*j*_ = *p*_*x*,*i*,*j*_/(1 − *p*_*x*,*i*,*j*_).

For human observers and for automatic classifiers, the predicted emotion is the emotion having the highest confidence score among the six emotions. By selecting the aggregated maximum confidence score as the indicator for emotion recognition, it is possible that more than one emotion label applies to the same video if they share identical overall confidence scores; in practice this occurred very rarely (S1 Fig).

A comparison is then performed between both the humans’ subjectively recognized emotion label and the automatic classifiers’ declared “recognized” emotion label with the corresponding emotion label for that facial expression [59]. If the predicted label (by human observers or automatic classifiers) matches the label assigned to the video, then the recognition is accurate. Otherwise, it is inaccurate. A detailed overview of the metrics for determining the confidence score, the recognized emotion label, and the emotion classification score per video is provided in S1 Table (human observers) and S2 Table (automatic classifiers). The analysis of recognition accuracy per video allows a comparison between the classifiers’ overall accuracy at a dataset level regardless of differences for specific emotions.

The classifiers’ pattern of accuracy can be evaluated by computing their Receiver Operating Characteristic (ROC) curve and the corresponding Area Under the Curve (AUC). The ROC curve and AUC values are obtained for each classifier by comparing the confidence score for the predicted label with its recognition accuracy (i.e., accurate recognition coded as 1 vs. inaccurate recognition coded as 0). In this context, the ROC curve is an indicator of the classifiers’ confidence in accurately recognizing an expression. A good classifier will accurately recognize expressions with high confidence and inaccurately recognize expressions with low confidence. In contrast, a poor classifier will inaccurately recognize expressions with high confidence and accurately recognize expressions with low confidence. The corresponding AUCs are the probability that a classifier will be more confident in accurately recognizing a facial expression. As such, the higher the AUC, the more confident the classifier is at accurately recognizing an expression.

## Results

Before assessing emotion classification in terms of recognition performance, we tested the interrater reliability of the multiple human observers and automatic classifiers involved in this study. Fleiss’ Kappa showed significant agreements in emotion ratings among the human observers (*κ* = 0.58, *p* < 0.001) and for the automatic classifiers (*κ* = 0.47, *p* < 0.001).

**Fig 1:**
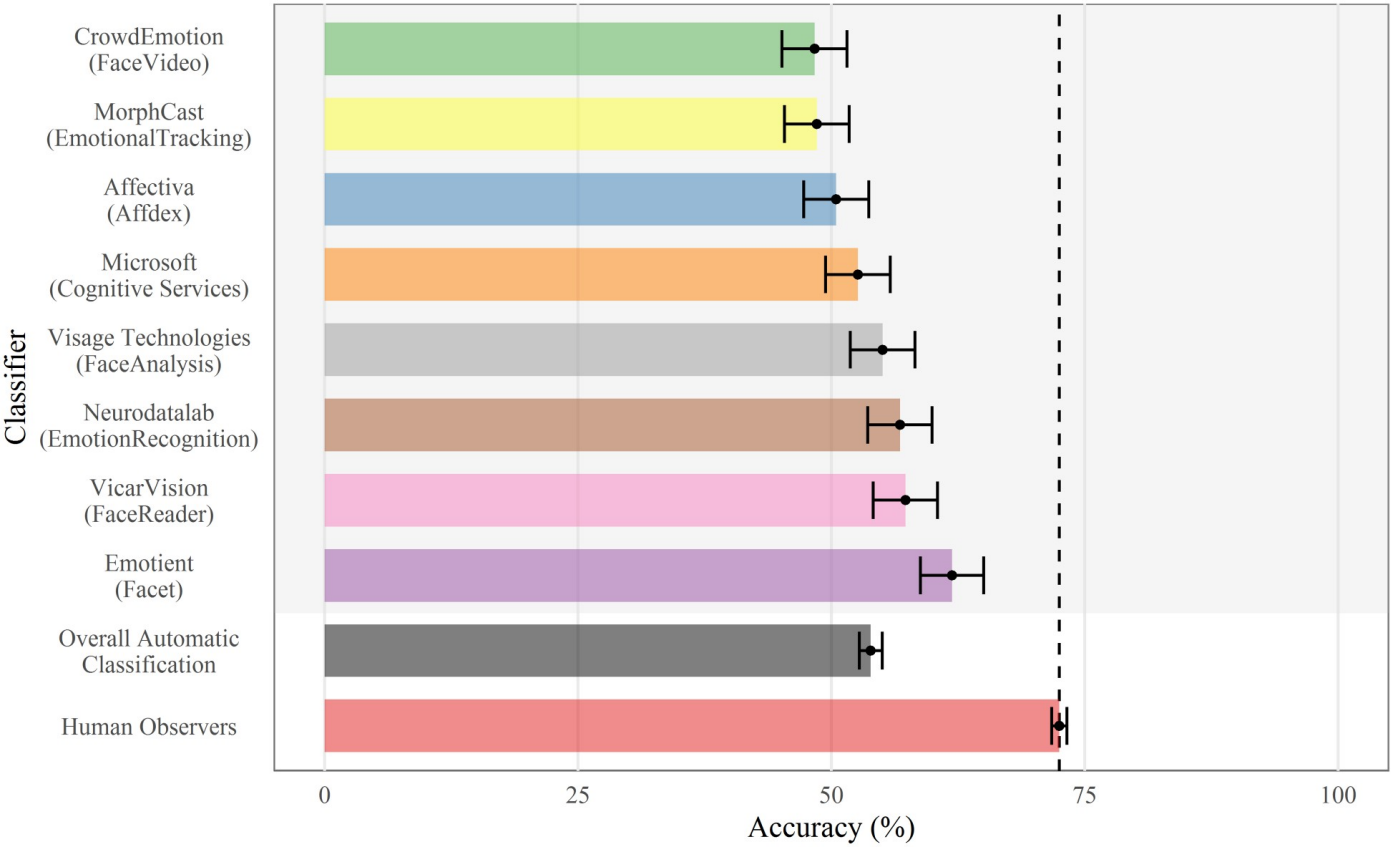
Mean True Positive recognition performance of human observers and automatic classifiers. Errors bars represent 95% Confidence Interval.

An analysis of the True Positive Rate (TPR) revealed that human observers generally performed better than the automatic classifiers (human observers: *M* = 72.48, 95%*CI* = [71.72;73.24] vs. automatic classifiers: *M* = 53.88, 95%*CI* = [52.75;55.01]). As can be seen in Fig 1, the best performance was obtained by Emotient (*M* = 61.9, 95%*CI* = [58.79;65.01]), followed by VicarVision (*M* = 57.31, 95%*CI* = [54.14;60.48]), Neurodata Lab (*M* = 56.78, 95%*CI* = [53.6;59.95]), Visage Technologies (*M* = 55.07, 95%*CI* = [51.88;58.26]), Microsoft (*M* = 52.61, 95%*CI* = [49.42;55.81]), Affectiva (*M* = 50.48, 95%*CI* = [47.28;53.68]), MorphCast (*M* = 48.56, 95%*CI* = [45.36;51.76]) and finally CrowdEmotion (*M* = 48.35, 95%*CI* = [45.14;51.55]).

### Recognition accuracy

**Fig 2:**
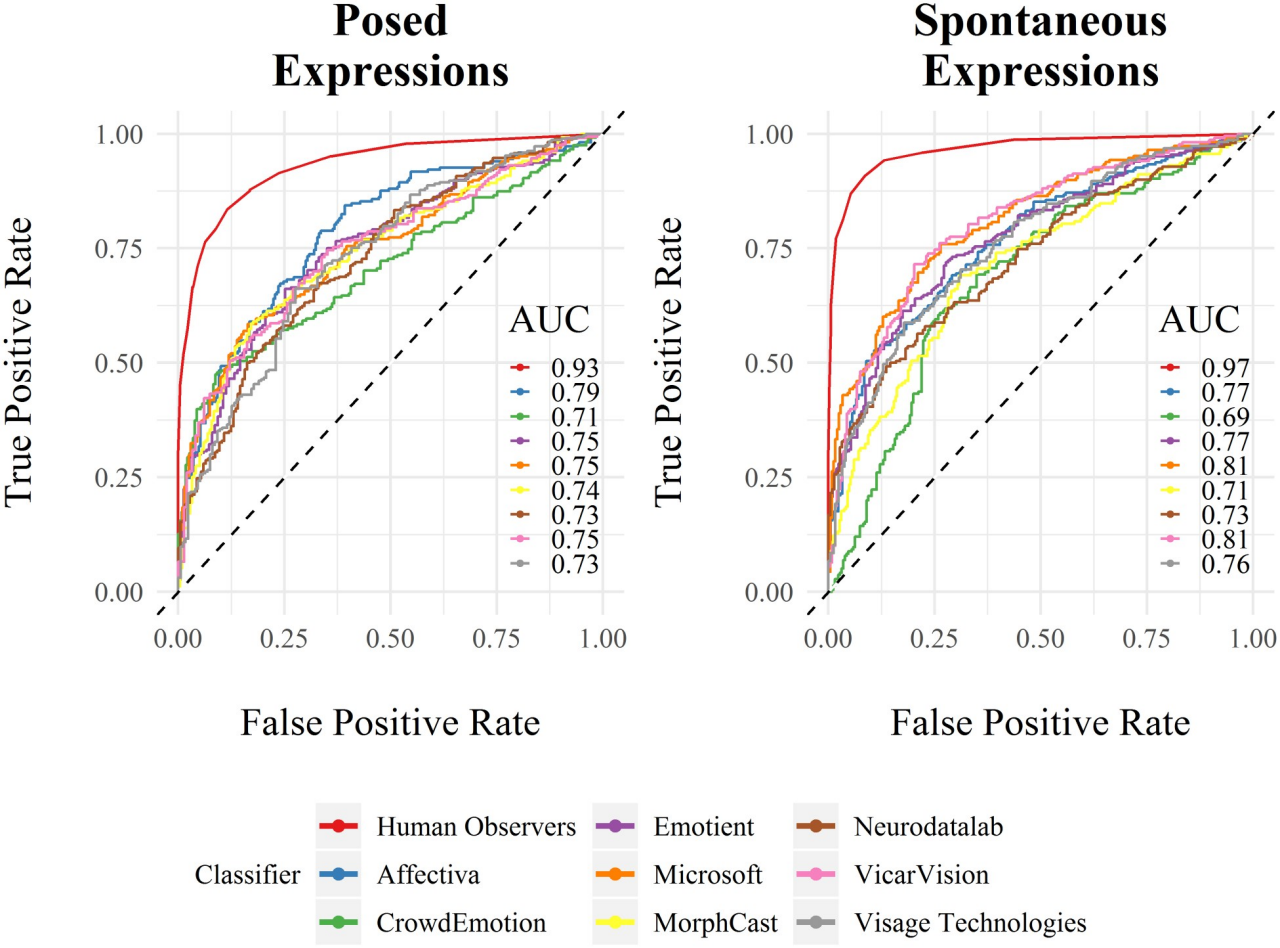
Receiver Operating Characteristic (ROC) curves and corresponding Area Under the Curve (AUC) depicting the True Positive Rate (TPR) against the False Positive Rate (FPR) for human observers and automatic classifiers separately for posed and spontaneous expressions. The dotted diagonal line in the ROC space indicates chance performance.

To further explore the classifiers’ diagnostic ability to discriminate between accurate and inaccurate recognition, ROC curves were plotted and the AUC was calculated. As illustrated in Fig 2, human observers exhibited the overall highest discrimination accuracy, with AUC values close to 1, thereby visibly outperforming all computer-based systems. The performance of the latter can be described as fair in the context of posed expressions. Interestingly, AUC scores were elevated in four out of the eight automatic classifiers when expressions were spontaneous. This is also exemplified by the steeper ROC curve in humans, indicating that the ability to accurately recognize facial expressions was facilitated by spontaneous affective displays. Because classification scores by human observers may vary with the number of observers under consideration, we further calculated the AUC scores for every combination of the 14 observers (see S2 Fig).

To compare the AUC from each classifier, pairwise two-sided bootstrap comparisons set to 2000 replications [60] were conducted (see S3 and S4 Tables for detailed Results). For each bootstrap replicate, the AUC of the two ROC curves are computed and the difference is stored. The following formula is used: *D* = (*AUC*1 − *AUC*2)/*s* where *s* is the standard deviation of the bootstrap differences and *AUC*1 and *AUC*2 are the AUC of the two (original) ROC curves. *D* is then compared to the normal distribution, according to the value of alternative. In the context of posed expressions, recognition rates by human observers had a significantly higher AUC compared to those of all other classifiers (*p*s < .001). Among the automatic classifiers, the pairwise AUC comparisons did not reveal any significant differences except between Affectiva and CrowdEmotion (*D*_*Af*−*CE*_ = 2.38, *p* = 0.017). The pattern of results was similar for spontaneous expressions, with a higher AUC for human observers in comparison to all other classifiers (*p*s < .001). Among the automatic classifiers, AUCs from Microsoft, VicarVison, Emotient, Affectiva and VisageTechnologies exceeded that from CrowdEmotion (*p*s < .05).

In addition to assessing the relative classification performance with ROC curves and their corresponding AUC [61], unweighted True Positive Rates (TPR), Positive Predicted Values (PPV), True Negative Rates (TNR) and F1 scores were calculated (see S6 and S5 Tables for detailed Results).

## Discussion

Following recent advances in automatic affect analysis, there has been a proliferation of commercially available automatic classifiers designed to recognize human facial expressions. Surprisingly, the number of independent peer-reviewed validation studies for these automatic classifiers is small and generally limited to validation using deliberately posed displays. The present study aimed to provide a multi-system evaluation of eight commercial automatic classifiers using two types of stimuli: posed expressions arising from instructions to portray a specific emotion, and spontaneous expressions in response to emotion-eliciting events. On the basis of dynamic stimuli sampled from two large databases, which differed on the described dimension of comparison, results revealed a recognition advantage for human observers over the automatic classifiers. The human recognition accuracy of 72% in the present study is consistent with evidence reported in the literature for dynamic expressions [62–64]. Among the eight classifiers tested in this work, we observed some variance in recognition accuracy, ranging from 48% to 62%.

Similar to past research [37, 38], recognition indices for the two best performing classifiers approximated those of human observers, suggesting high agreement in the classification of posed expressions. However, accuracy of most classifiers was consistently lower for spontaneous facial behavior. This could be due to the lack of prototypicality, that is, greater expressive variability, inherent in spontaneous affective responses. Because the emotional expression is induced via the presentation of emotion-eliciting materials, spontaneous displays have different properties than those that are deliberately instructed or enacted. For example, it has been shown that spontaneous facial actions differ in their temporal and morphological characteristics (e.g., duration, intensity, asymmetry) from posed ones [65]. Furthermore, the overall patterns of activity are often heterogeneous, which renders them more difficult to discern because of their ambiguous emotional content [66–68]. Results based on instructed and stereotypical facial portrayals may therefore not be directly transferable to those derived from activity occurring in spontaneous situations. Although dataset-specific features (i.e., uneven distributions of spontaneous stimuli across the six emotion categories) might independently affect emotion recognition, both types of stimuli were recorded under relatively controlled experimental conditions.

This conclusion further appears to be supported by the observed similarity in patterns of confusion errors between humans and the automatic classifiers. While the present results suggested considerable overlap in the type of confusions for posed expressions, these correlations were much weaker in the case of spontaneous expressions. Further analyses showed that discrimination accuracy (i.e., the AUC) was on average lower for all eight automatic classifiers. When comparing AUC values, human observers clearly outperformed all automatic classifiers in recognizing emotions from both spontaneous and posed expressions. The results did not reveal any significant differences between the eight automatic classifiers (except when comparing CrowdEmotion to the other classifiers in the context of spontaneous facial expressions). Thus, the manner in which affective information is automatically extracted is almost certainly not the same compared to how human observers achieve the task [29, 69]. Such discrepancies can likely be explained by the quality and quantity of data available to train computer-based systems. Although several efforts have been reported over the last few years on the automatic analysis of spontaneous displays [55, 70], most current automatic classifiers have typically been trained and tested using posed or acted facial behavior. Besides their limited ability to transfer to the subtlety and complexity of spontaneous recordings [43], the highly standardized form of prototypical expressions makes it difficult to generalize beyond specific training sets.

At the technical level, the problem of over-fitting is likely to be prevalent. That is, the classifiers may have learned to respond too closely to artificially uniform training sets, thereby losing flexibility when they are applied to unexpectedly subtle and ambiguous expressions. To develop more robust models in the future, it will be important to obtain and train on more databases that display spontaneous and even naturalistic behavior [34]. The latter type of behavior denotes affective responses recorded in real-life settings (i.e., “in the wild”). Because naturalistic expressions are not elicited in the laboratory, they are the least experimentally controlled [42]; as such, they have multiple social functions and are driven by a variety of socio-cultural and contextual influences. To achieve this aim, metadata in the form of self-reports, behavioral coding [71], and physiological (facial EMG), or neuroscientific measures (EEG, fMRI) are needed to specify the emotional content of recordings. Such annotation of large video sets can help accelerate the progress of affective computing research by providing more comprehensive benchmarks for the training and testing of automatic classifiers on spontaneous expressions.

While BET is the most commonly used taxonomy in affective computing, it must be noted that such a perspective is unlikely to reflect the full range of everyday emotions. Typically, emotional behavior “in the wild” involves a wide variety of affective displays that span a substantial number of emotional states beyond the basic six. Even if this may include prototypical AU configurations, emotion expressions are likely to vary across cultures, contexts and individuals [9]. Also, one cannot assume a one-to-one correspondence between the experience and expression of emotion [28]. Given that facial expressions fulfill a range of functions (e.g., appraisals, action tendencies, social motives), it is unlikely that they always signal current emotions in the sense of a “readout” [3, 17]. Just because a person is smiling does not mean that s/he is happy. Computer-based systems using the BET perspective to detect discrete emotions from facial displays may therefore stand on questionable theoretical and empirical grounds. Also, expressions span a large range of psychological phenomena. To account for this complexity, a few tentative efforts in computer vision have recently started to address non-basic affective and mental states such as interest, pain, boredom, and frustration [72, 73]. By extending the number of emotion categories, automated methods might overcome their current limitation of classifying a small set of emotion labels that are insufficient to describe the complexity of human expressive behaviors. Consequently, we may be able to gain a fuller understanding of the signals and functions of affective phenomena in the future.

Prospective approaches to automatic classification of human affect should further aim to integrate relevant contextual information, as well as learn to better suppress irrelevant information. Both databases used in this work comprised stimuli recorded under relatively controlled conditions, and depicted full frontal shots with neutral backgrounds and steady head poses. While these databases have kept contextual variations across senders constant, information about the wider physical environment and situational factors is likely to be critical to human perception outside the laboratory. Apart from the present limitation of using only two datasets, this would also make the stimuli more representative of the situations in which classifiers are actually employed. Past research, for example, has shown that the same facial expression is interpreted differently depending on the social context in which it occurs [74, 75]. Moreover, context helps to disambiguate between various exemplars of an emotion category [76]. Failures to address the relative role of context may therefore lead to difficulties in classification processes generalizing to real-world settings with natural expressions. Issues regarding the poor generalization capacity of machine analyses have recently led to a call for new regulations in the use of affective computing technologies, especially when applied to organizational and decision-making processes [77]. It will fall to future research to train and test relevant computer systems on more ecologically valid and meaningful materials that are representative of a wider range of emotional and situational contexts. The present study is a first attempt to provide a systematic multi-system evaluation of current commercial automatic classifiers using the basic six emotions. By doing so, we hope to help pave the way for the development of more robust automatic classifiers in the future.

## Supporting information

S1 Table Metrics for determining the confidence score (CS), the recognized emotion label, and the emotion classification score per video (B) based on the raw data (A) from human observers. (PDF)

S2 Table Metrics for determining the confidence score (CS), the recognized emotion label, and the emotion classification score per video (B) based on the raw data (A) from the automatic classifiers. (PDF)

S3 Table Pairwise two-sided bootstrap comparision of the Receiver Operating Characteristic (ROC)’s Area Under the Curve (AUC) between the classifiers for posed facial expressions. (PDF)

S4 Table Pairwise two-sided bootstrap comparision of the Receiver Operating Characteristic (ROC)’s Area Under the Curve (AUC) between the classifiers for spontaneous expressions. (PDF)

S5 Table Performance indices for human observers and automatic classifiers by emotion in the context of posed expressions. (PDF)

S6 Table Performance indices for human observers and automatic classifiers by emotion in the context of spontaneous expressions. (PDF)

S1 Fig Emotion confusion matrices for human observers and automatic classifiers separately by posed and spontaneous expressions. For cases with ‘undetermined’ confidence levels, the sum of the marginal proportion of recognized emotions can be higher than 100%.
(PDF)

S2 Fig Mean values and standard deviations of the Area Under the Curve (AUC) by type of expression for every combination of the 14 human observers. (PDF)
