# Supplementary material for: A performance comparison of eight commercially available automatic classifiers for facial affect recognition
Source: PLoS One. 2020 Apr 24;15(4):e0231968. doi: 10.1371/journal.pone.0231968 (PMC7182192; doi:10.1371/journal.pone.0231968)
Supplement: S1 Table — (PDF) [file pone.0231968.s001.pdf]

S1 Table. Metrics for determining the confidence score (CS), the recognized emotion label, and the emotion classification score per video (B) based on the raw data (A) from human observers.

A

$Video$	$EmotionDisplayed$	$HunanObserver_1$	$\dots$	$HunanObserver_k$
$Video_1$	$EmoDis_{1,1}$	$EmoRec_{1,1,1}$	$\dots$	$EmoRec_{1,j,k}$
$Video_2$	$EmoDis_{2,2}$	$EmoRec_{2,2,1}$	$\dots$	$EmoRec_{2,j,k}$
$\vdots$	$\vdots$	$\vdots$	$\vdots$	$\vdots$
$Video_i$	$EmoDis_{i,j}$	$EmoRec_{i,j,1}$	$\dots$	$EmoRec_{i,j,k}$

B

$Video$	$EmotionDisplayed$	$CSEmotionRecognized_1$	$\dots$	$CSEmotionRecognized_j$	$EmotionRecognized$	$EmotionClassification$
$Video_1$	$EmoDis_{1,1}$	$\frac{1}{K}\sum_{k=1}^{K} EmoRec_{1,1,k}$	$\dots$	$\frac{1}{K}\sum_{k=1}^{K} EmoRec_{1,j,k}$	$\max(CSEmoRec_{1,j})$	$\begin{cases} EmoDis_{1,1} = EmoRec_{1,j}, 1 \\ EmoDis_{1,1} \neq EmoRec_{1,j}, 0 \end{cases}$
$Video_2$	$EmoDis_{2,2}$	$\frac{1}{K}\sum_{k=1}^{K} EmoRec_{2,1,k}$	$\dots$	$\frac{1}{K}\sum_{k=1}^{K} EmoRec_{2,j,k}$	$\max(CSEmoRec_{2,j})$	$\begin{cases} EmoDis_{2,2} = EmoRec_{2,j}, 1 \\ EmoDis_{2,2} \neq EmoRec_{2,j}, 0 \end{cases}$
$\vdots$	$\vdots$	$\vdots$	$\vdots$	$\vdots$	$\vdots$	$\vdots$
$Video_i$	$EmoDis_{i,j}$	$\frac{1}{K}\sum_{k=1}^{K} EmoRec_{i,1,k}$	$\dots$	$\frac{1}{K}\sum_{k=1}^{K} EmoRec_{i,j,k}$	$\max(CSEmoRec_{i,j})$	$\begin{cases} EmoDis_{i,j} = EmoRec_{i,j}, 1 \\ EmoDis_{i,j} \neq EmoRec_{i,j}, 0 \end{cases}$

Note: i refers to one of the 937 videos processed, j is an emotion label among *anger*, *disgust*, *fear*, *happiness*, *sadness* and *surprise*, and k is one of the 14 human observers.
