# Supplementary material for: A performance comparison of eight commercially available automatic classifiers for facial affect recognition
Source: PLoS One. 2020 Apr 24;15(4):e0231968. doi: 10.1371/journal.pone.0231968 (PMC7182192; doi:10.1371/journal.pone.0231968)
Supplement: S2 Table — (PDF) [file pone.0231968.s002.pdf]

S2 Table. Metrics for determining the confidence score (CS), the recognized emotion label, and the emotion classification score per video (B) based on the raw data (A) from the automatic classifiers.

A

$Video$	$Frame$	$EmotionDisplayed$	$EmotionRecognized_1$	$\dots$	$EmotionRecognized_j$
$Video_1$	t_0	$EmoDis_{1,1}$	$\psi_{0,1,1}$	$\dots$	$\psi_{0,1,j}$
$Video_1$	t_1	$EmoDis_{1,1}$	$\psi_{1,1,1}$	$\dots$	$\psi_{1,1,j}$
$Video_1$	$\vdots$	$\vdots$	$\vdots$	$\vdots$	$\vdots$
$Video_1$	t_x	$EmoDis_{1,1}$	$\psi_{x,1,1}$	$\dots$	$\psi_{x,1,j}$
$Video_2$	t_0	$EmoDis_{2,2}$	$\psi_{0,2,1}$	$\dots$	$\psi_{0,2,j}$
$Video_2$	t_1	$EmoDis_{2,2}$	$\psi_{1,2,1}$	$\dots$	$\psi_{1,2,j}$
$Video_2$	$\vdots$	$\vdots$	$\vdots$	$\vdots$	$\vdots$
$Video_2$	t_x	$EmoDis_{2,2}$	$\psi_{x,2,1}$	$\dots$	$\psi_{x,2,j}$
$\vdots$	$\vdots$	$\vdots$	$\vdots$	$\vdots$	$\vdots$
$Video_i$	t_0	$EmoDis_{i,j}$	$\psi_{0,i,1}$	$\dots$	$\psi_{0,i,j}$
$Video_i$	t_1	$EmoDis_{i,j}$	$\psi_{1,i,1}$	$\dots$	$\psi_{1,i,j}$
$Video_i$	$\vdots$	$\vdots$	$\vdots$	$\vdots$	$\vdots$
$Video_i$	t_x	$EmoDis_{i,j}$	$\psi_{x,i,1}$	$\dots$	$\psi_{x,i,j}$

B

$Video$	$EmotionDisplayed$	$CSEmotionRecognized_1$	$\dots$	$CSEmotionRecognized_j$	$EmotionRecognized$	$EmotionClassification$
$Video_1$	$EmoDis_{1,1}$	$\frac{\sum_{x=0}^{T}\psi_{x,1,1}}{\sum_{j=1}^{J}\sum_{x=0}^{T}\psi_{x,1,j}}$	$\dots$	$\frac{\sum_{x=0}^{T}\psi_{x,1,j}}{\sum_{j=1}^{J}\sum_{x=0}^{T}\psi_{x,1,j}}$	$\max(CSEmoRec_{1,j})$	$\begin{cases} EmoDis_{1,1} = EmoRec_{1,j}, 1 \\ EmoDis_{1,1} \neq EmoRec_{1,j}, 0 \end{cases}$
$Video_2$	$EmoDis_{2,2}$	$\frac{\sum_{x=0}^{T}\psi_{x,2,1}}{\sum_{j=1}^{J}\sum_{x=0}^{T}\psi_{x,2,j}}$	$\dots$	$\frac{\sum_{x=0}^{T}\psi_{x,2,j}}{\sum_{j=1}^{J}\sum_{x=0}^{T}\psi_{x,2,j}}$	$\max(CSEmoRec_{2,j})$	$\begin{cases} EmoDis_{2,2} = EmoRec_{2,j}, 1 \\ EmoDis_{2,2} \neq EmoRec_{2,j}, 0 \end{cases}$
$\vdots$	$\vdots$	$\vdots$	$\vdots$	$\vdots$	$\vdots$	$\vdots$
$Video_i$	$EmoDis_{i,j}$	$\frac{\sum_{x=0}^{T}\psi_{x,i,1}}{\sum_{j=1}^{J}\sum_{x=0}^{T}\psi_{x,i,j}}$	$\dots$	$\frac{\sum_{x=0}^{T}\psi_{x,i,j}}{\sum_{j=1}^{J}\sum_{x=0}^{T}\psi_{x,i,j}}$	$\max(CSEmoRec_{i,j})$	$\begin{cases} EmoDis_{i,j} = EmoRec_{i,j}, 1 \\ EmoDis_{i,j} \neq EmoRec_{i,j}, 0 \end{cases}$

Note: i is one of the 937 videos processed, x is a video frame at t_x, j is an emotion label among *anger*, *disgust*, *fear*, *happiness*, *sadness* and *surprise*, and ψ is the value of the odds ratio for the frame t_x and for the emotion label j.
