# Supplementary material for: A performance comparison of eight commercially available automatic classifiers for facial affect recognition
Source: PLoS One. 2020 Apr 24;15(4):e0231968. doi: 10.1371/journal.pone.0231968 (PMC7182192; doi:10.1371/journal.pone.0231968)
Supplement: S3 Table — (PDF) [file pone.0231968.s003.pdf]

S3 Table. Pairwise two-sided bootstrap comparision of the Receiver Operating Characteristic (ROC)'s Area Under the Curve (AUC) between the classifiers for posed facial expressions.

classifier1	classifier2	database	auc1	auc2	d_stat	p_val
Affectiva	CrowdEmotion	Posed	0.79	0.71	2.38	0.017
Affectiva	Emotient	Posed	0.79	0.75	1.07	0.285
Affectiva	Microsoft	Posed	0.79	0.75	1.18	0.238
Affectiva	MorphCast	Posed	0.79	0.74	1.44	0.15
Affectiva	Neurodatalab	Posed	0.79	0.73	1.68	0.094
Affectiva	VicarVision	Posed	0.79	0.75	1.21	0.226
Affectiva	VisageTechnologies	Posed	0.79	0.73	1.70	0.09
CrowdEmotion	Emotient	Posed	0.71	0.75	-1.35	0.178
CrowdEmotion	Microsoft	Posed	0.71	0.75	-1.27	0.203
CrowdEmotion	MorphCast	Posed	0.71	0.74	-1.06	0.29
CrowdEmotion	Neurodatalab	Posed	0.71	0.73	-0.73	0.468
CrowdEmotion	VicarVision	Posed	0.71	0.75	-1.22	0.221
CrowdEmotion	VisageTechnologies	Posed	0.71	0.73	-0.75	0.452
Emotient	Microsoft	Posed	0.75	0.75	0.08	0.934
Emotient	MorphCast	Posed	0.75	0.74	0.30	0.768
Emotient	Neurodatalab	Posed	0.75	0.73	0.64	0.525
Emotient	VicarVision	Posed	0.75	0.75	0.12	0.902
Emotient	VisageTechnologies	Posed	0.75	0.73	0.56	0.573
Humans	Affectiva	Posed	0.93	0.79	6.68	< 0.001
Humans	CrowdEmotion	Posed	0.93	0.71	9.01	< 0.001
Humans	Emotient	Posed	0.93	0.75	7.31	< 0.001
Humans	Microsoft	Posed	0.93	0.75	7.70	< 0.001
Humans	MorphCast	Posed	0.93	0.74	7.92	< 0.001
Humans	Neurodatalab	Posed	0.93	0.73	8.08	< 0.001
Humans	VicarVision	Posed	0.93	0.75	7.43	< 0.001
Humans	VisageTechnologies	Posed	0.93	0.73	7.90	< 0.001
Microsoft	MorphCast	Posed	0.75	0.74	0.22	0.829
Microsoft	Neurodatalab	Posed	0.75	0.73	0.55	0.582
Microsoft	VicarVision	Posed	0.75	0.75	0.04	0.968
Microsoft	VisageTechnologies	Posed	0.75	0.73	0.49	0.627
MorphCast	Neurodatalab	Posed	0.74	0.73	0.34	0.731
MorphCast	VicarVision	Posed	0.74	0.75	-0.17	0.865
MorphCast	VisageTechnologies	Posed	0.74	0.73	0.28	0.783
Neurodatalab	VicarVision	Posed	0.73	0.75	-0.50	0.614
Neurodatalab	VisageTechnologies	Posed	0.73	0.73	-0.06	0.953
VicarVision	VisageTechnologies	Posed	0.75	0.73	0.45	0.654
