# Supplementary material for: A performance comparison of eight commercially available automatic classifiers for facial affect recognition
Source: PLoS One. 2020 Apr 24;15(4):e0231968. doi: 10.1371/journal.pone.0231968 (PMC7182192; doi:10.1371/journal.pone.0231968)
Supplement: S4 Table — (PDF) [file pone.0231968.s004.pdf]

S4 Table. Pairwise two-sided bootstrap comparision of the Receiver Operating Characteristic (ROC)'s Area Under the Curve (AUC) between the classifiers for spontaneous expressions.

classifier1	classifier2	database	auc1	auc2	d__stat	p__val
Affectiva	CrowdEmotion	Spontaneous	0.77	0.69	2.46	0.014
Affectiva	Emotient	Spontaneous	0.77	0.77	-0.15	0.879
Affectiva	Microsoft	Spontaneous	0.77	0.81	-1.35	0.176
Affectiva	MorphCast	Spontaneous	0.77	0.71	1.74	0.081
Affectiva	Neurodatalab	Spontaneous	0.77	0.73	1.32	0.188
Affectiva	VicarVision	Spontaneous	0.77	0.81	-1.23	0.219
Affectiva	VisageTechnologies	Spontaneous	0.77	0.76	0.25	0.799
CrowdEmotion	Emotient	Spontaneous	0.69	0.77	-2.63	0.009
CrowdEmotion	Microsoft	Spontaneous	0.69	0.81	-3.82	< 0.001
CrowdEmotion	MorphCast	Spontaneous	0.69	0.71	-0.74	0.462
CrowdEmotion	Neurodatalab	Spontaneous	0.69	0.73	-1.20	0.232
CrowdEmotion	VicarVision	Spontaneous	0.69	0.81	-3.58	< 0.001
CrowdEmotion	VisageTechnologies	Spontaneous	0.69	0.76	-2.24	0.025
Emotient	Microsoft	Spontaneous	0.77	0.81	-1.23	0.218
Emotient	MorphCast	Spontaneous	0.77	0.71	1.91	0.056
Emotient	Neurodatalab	Spontaneous	0.77	0.73	1.43	0.151
Emotient	VicarVision	Spontaneous	0.77	0.81	-1.05	0.296
Emotient	VisageTechnologies	Spontaneous	0.77	0.76	0.42	0.678
Humans	Affectiva	Spontaneous	0.97	0.77	8.98	< 0.001
Humans	CrowdEmotion	Spontaneous	0.97	0.69	10.92	< 0.001
Humans	Emotient	Spontaneous	0.97	0.77	8.55	< 0.001
Humans	Microsoft	Spontaneous	0.97	0.81	7.58	< 0.001
Humans	MorphCast	Spontaneous	0.97	0.71	10.12	< 0.001
Humans	Neurodatalab	Spontaneous	0.97	0.73	10.34	< 0.001
Humans	VicarVision	Spontaneous	0.97	0.81	7.49	< 0.001
Humans	VisageTechnologies	Spontaneous	0.97	0.76	9.18	< 0.001
Microsoft	MorphCast	Spontaneous	0.81	0.71	3.19	0.001
Microsoft	Neurodatalab	Spontaneous	0.81	0.73	2.73	0.006
Microsoft	VicarVision	Spontaneous	0.81	0.81	0.17	0.863
Microsoft	VisageTechnologies	Spontaneous	0.81	0.76	1.63	0.104
MorphCast	Neurodatalab	Spontaneous	0.71	0.73	-0.48	0.628
MorphCast	VicarVision	Spontaneous	0.71	0.81	-2.89	0.004
MorphCast	VisageTechnologies	Spontaneous	0.71	0.76	-1.51	0.13
Neurodatalab	VicarVision	Spontaneous	0.73	0.81	-2.51	0.012
Neurodatalab	VisageTechnologies	Spontaneous	0.73	0.76	-1.03	0.303
VicarVision	VisageTechnologies	Spontaneous	0.81	0.76	1.45	0.148
