# Supplementary material for: A performance comparison of eight commercially available automatic classifiers for facial affect recognition
Source: PLoS One. 2020 Apr 24;15(4):e0231968. doi: 10.1371/journal.pone.0231968 (PMC7182192; doi:10.1371/journal.pone.0231968)
Supplement: S5 Table — (PDF) [file pone.0231968.s005.pdf]

S5 Table. Performance indices for human observers and automatic classifiers by emotion in the context of posed expressions.

Emotion	TPR	PPV	TNR	F1
**Human Observers**				
anger	0.68	0.66	0.93	0.67
disgust	0.64	0.55	0.91	0.59
fear	0.73	0.52	0.91	0.61
happiness	0.92	0.97	0.99	0.94
sadness	0.90	0.86	0.97	0.88
surprise	0.84	0.87	0.97	0.86
**Affectiva**				
anger	0.60	0.23	0.86	0.33
disgust	0.29	0.67	0.91	0.40
fear	0.41	0.17	0.85	0.24
happiness	0.68	0.83	0.97	0.75
sadness	0.47	0.32	0.87	0.38
surprise	0.57	0.57	0.92	0.57
**CrowdEmotion**				
anger	0.54	0.35	0.88	0.42
disgust	0.38	0.50	0.89	0.43
fear	0.67	0.10	0.85	0.18
happiness	0.50	0.97	0.99	0.66
sadness	0.60	0.31	0.87	0.41
surprise	0.59	0.83	0.96	0.69
**Emotient**				
anger	0.72	0.55	0.91	0.62
disgust	0.56	0.77	0.95	0.65
fear	0.95	0.26	0.87	0.40
happiness	0.61	0.96	0.99	0.75
sadness	0.74	0.67	0.93	0.70
surprise	0.77	0.86	0.97	0.81
**Microsoft**				
anger	0.56	0.28	0.87	0.38
disgust	0.65	0.36	0.88	0.46
fear	1.00	0.06	0.84	0.12
happiness	0.47	1.00	1.00	0.64
sadness	0.53	0.82	0.96	0.64
surprise	0.72	0.88	0.98	0.80
**MorphCast**				
anger	0.36	0.26	0.86	0.30
disgust	0.53	0.45	0.89	0.49
fear	0.38	0.33	0.87	0.36
happiness	0.67	0.87	0.97	0.76
sadness	0.51	0.58	0.91	0.54
surprise	0.65	0.74	0.95	0.69
**Neurodata Lab**				
anger	0.57	0.26	0.87	0.35
disgust	0.53	0.68	0.93	0.60
fear	0.48	0.18	0.85	0.26
happiness	0.78	0.95	0.99	0.86
sadness	0.49	0.79	0.95	0.61
surprise	0.72	0.77	0.95	0.74
**VicarVision**				
anger	0.60	0.44	0.89	0.50
disgust	0.59	0.65	0.93	0.62
fear	0.76	0.36	0.88	0.49
happiness	0.73	0.96	0.99	0.83
sadness	0.62	0.85	0.97	0.72
surprise	0.84	0.84	0.97	0.84
**Visage Technologies**				
anger	0.55	0.50	0.90	0.52
disgust	0.63	0.53	0.91	0.57
fear	0.59	0.24	0.86	0.35
happiness	0.62	0.95	0.99	0.75
sadness	0.60	0.76	0.95	0.67
surprise	0.75	0.79	0.96	0.77
