# Supplementary material for: A performance comparison of eight commercially available automatic classifiers for facial affect recognition
Source: PLoS One. 2020 Apr 24;15(4):e0231968. doi: 10.1371/journal.pone.0231968 (PMC7182192; doi:10.1371/journal.pone.0231968)
Supplement: S6 Table — (PDF) [file pone.0231968.s006.pdf]

S6 Table. Performance indices for human observers and automatic classifiers by emotion in the context of spontaneous expressions.

Emotion	TPR	PPV	TNR	F1
**Human Observers**				
anger	0.08	0.52	1.00	0.14
disgust	0.89	0.60	0.88	0.71
fear	0.11	0.26	0.98	0.15
happiness	0.97	0.83	0.89	0.89
sadness	0.54	0.68	0.97	0.60
surprise	0.81	0.68	0.92	0.74
**Affectiva**				
anger	0.00	0.00	0.99	
disgust	0.39	0.44	0.80	0.41
fear	0.09	0.23	0.98	0.13
happiness	0.75	0.78	0.84	0.76
sadness	0.19	0.13	0.93	0.15
surprise	0.77	0.43	0.86	0.55
**CrowdEmotion**				
anger	0.00	0.00	0.99	
disgust	0.36	0.29	0.78	0.33
fear	0.15	0.15	0.98	0.15
happiness	0.63	0.80	0.82	0.70
sadness	0.24	0.42	0.95	0.30
surprise	0.33	0.06	0.79	0.10
**Emotient**				
anger	0.02	0.33	1.00	0.04
disgust	0.83	0.38	0.82	0.52
fear	0.22	0.31	0.98	0.26
happiness	0.70	0.93	0.93	0.80
sadness	0.32	0.58	0.96	0.42
surprise	0.59	0.10	0.80	0.17
**Microsoft**				
anger	0.03	0.33	1.00	0.05
disgust	1.00	0.04	0.75	0.08
fear		0.00	0.97	
happiness	0.63	0.98	0.98	0.77
sadness	0.21	0.66	0.96	0.32
surprise	0.50	0.05	0.79	0.09
**MorphCast**				
anger	0.03	0.33	1.00	0.05
disgust	0.51	0.46	0.82	0.49
fear	0.05	0.31	0.98	0.08
happiness	0.78	0.59	0.75	0.67
sadness	0.24	0.47	0.95	0.32
surprise	0.58	0.11	0.80	0.18
**Neurodata Lab**				
anger	0.08	1.00	1.00	0.14
disgust	0.70	0.41	0.82	0.52
fear	0.15	0.15	0.98	0.15
happiness	0.79	0.83	0.88	0.81
sadness	0.21	0.76	0.97	0.33
surprise	1.00	0.04	0.79	0.08
**VicarVision**				
anger	0.01	0.33	0.99	0.02
disgust	0.82	0.19	0.78	0.31
fear	0.05	0.15	0.97	0.08
happiness	0.75	0.80	0.85	0.77
sadness	0.27	0.66	0.97	0.38
surprise	0.69	0.11	0.80	0.19
**Visage Technologies**				
anger	0.02	0.33	1.00	0.03
disgust	0.79	0.26	0.80	0.39
fear	0.06	0.08	0.97	0.07
happiness	0.71	0.85	0.87	0.77
sadness	0.20	0.61	0.96	0.30
surprise	0.25	0.01	0.79	0.02
