# Supplementary material for: A performance comparison of eight commercially available automatic classifiers for facial affect recognition
Source: PLoS One. 2020 Apr 24;15(4):e0231968. doi: 10.1371/journal.pone.0231968 (PMC7182192; doi:10.1371/journal.pone.0231968)
Supplement: S1 Fig — For cases with ‘undetermined’ confidence levels, the sum of the marginal proportion of recognized emotions can be higher than 100%. (PDF) [file pone.0231968.s007.pdf]

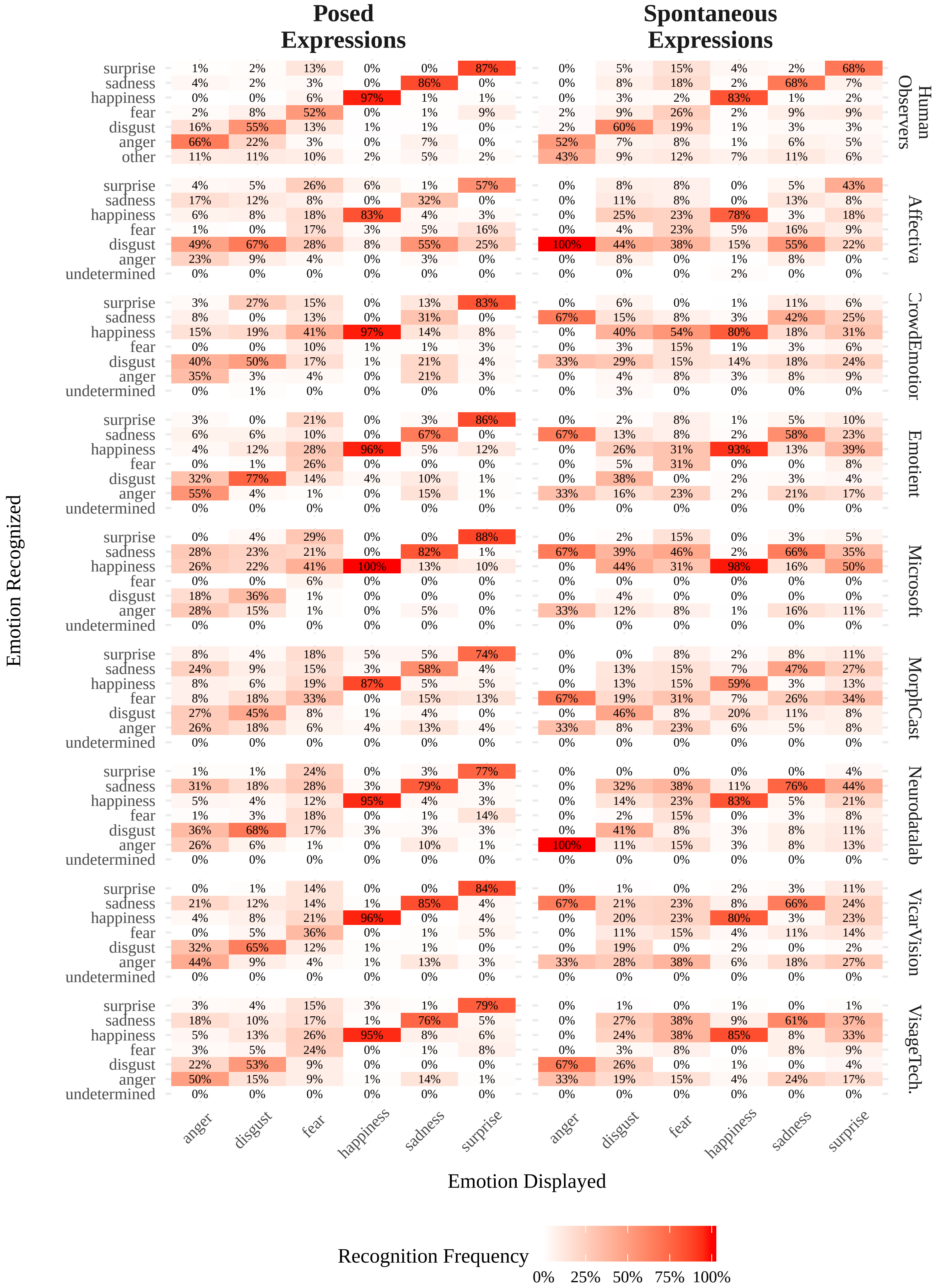

S1 Figure. Emotion confusion matrices for human observers and automatic classifiers separately by posed and spontaneous expressions. For cases with ‘undetermined’ confidence levels, the sum of the marginal proportion of recognized emotions can be higher than 100%.
