# Supplementary material for: A performance comparison of eight commercially available automatic classifiers for facial affect recognition
Source: PLoS One. 2020 Apr 24;15(4):e0231968. doi: 10.1371/journal.pone.0231968 (PMC7182192; doi:10.1371/journal.pone.0231968)
Supplement: S2 Fig — (PDF) [file pone.0231968.s008.pdf]

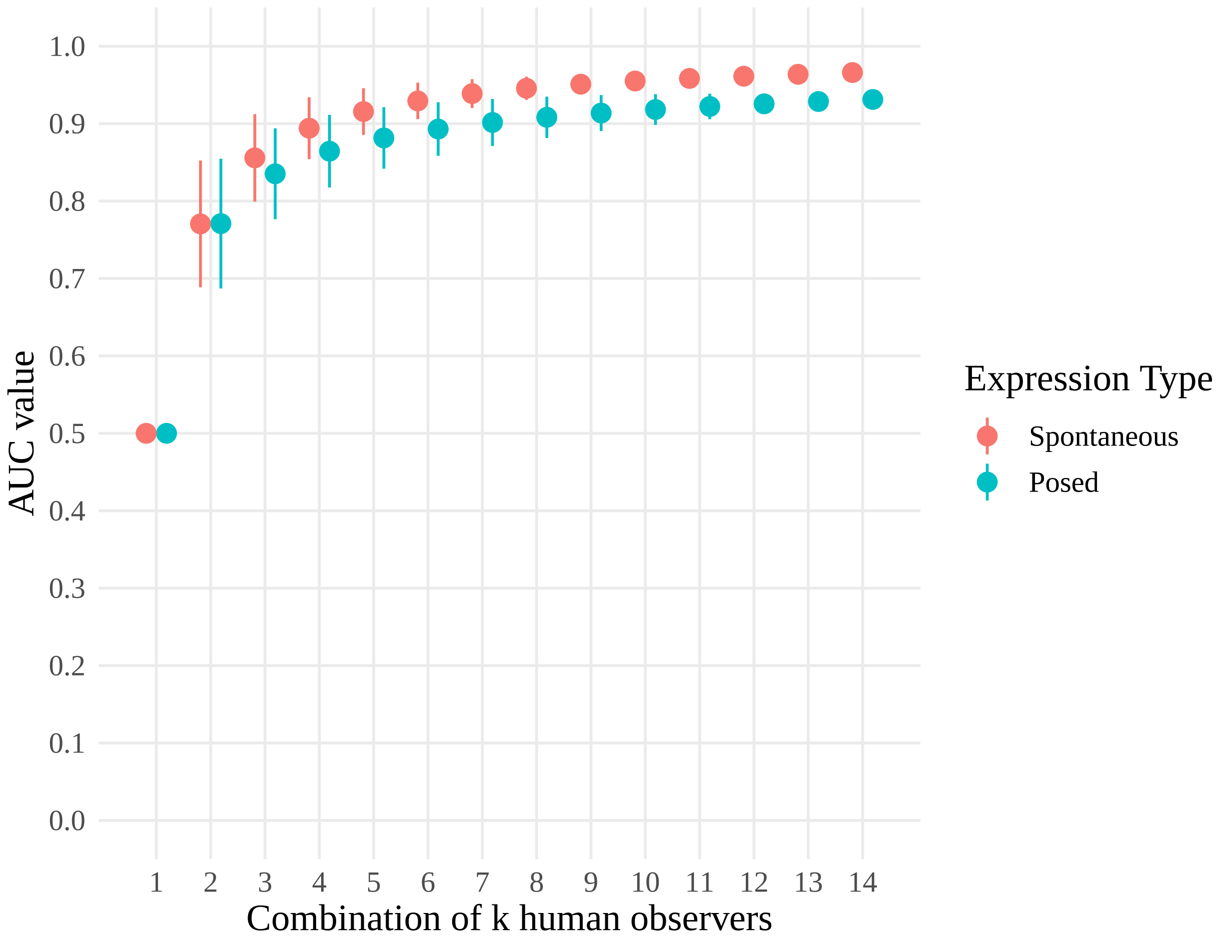

S2 Figure. Mean values and standard deviations of the Area Under the Curve (AUC) by type of expression for every combination of the 14 human observers.
